# Influence of Anthropogenic Climate Change on Planetary Wave Resonance and Extreme Weather Events

**DOI:** 10.1038/srep45242

**Published:** 2017-03-27

**Authors:** Michael E. Mann, Stefan Rahmstorf, Kai Kornhuber, Byron A. Steinman, Sonya K. Miller, Dim Coumou

**Affiliations:** 1 Department of Meteorology and Atmospheric Science, Pennsylvania State University, University Park, PA USA; 2 Earth System Analysis, Potsdam Institute for Climate Impact Research, Potsdam, Germany; 3 Department of Earth and Environmental Sciences and Large Lakes Observatory, University of Minnesota Duluth, Duluth, Minnesota, USA; 4 Institute for Environmental Studies (IVM), VU University Amsterdam, Amsterdam, The Netherlands.

## Abstract

Persistent episodes of extreme weather in the Northern Hemisphere summer have been shown to be associated with the presence of high-amplitude quasi-stationary atmospheric Rossby waves within a particular wavelength range (zonal wavenumber 6–8). The underlying mechanistic relationship involves the phenomenon of quasi-resonant amplification (QRA) of synoptic-scale waves with that wavenumber range becoming trapped within an effective mid-latitude atmospheric waveguide. Recent work suggests an increase in recent decades in the occurrence of QRA-favorable conditions and associated extreme weather, possibly linked to amplified Arctic warming and thus a climate change influence. Here, we isolate a specific fingerprint in the zonal mean surface temperature profile that is associated with QRA-favorable conditions. State-of-the-art (“CMIP5”) historical climate model simulations subject to anthropogenic forcing display an increase in the projection of this fingerprint that is mirrored in multiple observational surface temperature datasets. Both the models and observations suggest this signal has only recently emerged from the background noise of natural variability.

A series of persistent, extreme summer weather events in recent years including the 2003 European Heat Wave, the 2010 Pakistan flood/Russian heatwave, 2011 Texas drought and the unprecedented, ongoing drought in California, has led to a continuing discussion in the scientific literature regarding the relationship between anthropogenic climate change and the spate of recent weather extremes^1^,^2^,^3^,^4^,^5^,^6^,^7^,^8^.

Some of the increase in extreme events can be explained by relatively straightforward thermodynamics, wherein modest shifts in mean temperature lead to increases in the frequency of heatwaves^9^,^10^,^11^,^12^ or wherein rising temperatures favor more intense precipitation events via moist thermodynamics^13^,^14^,^15^. However, a growing number of studies suggests that these mechanisms alone are not sufficiently explanatory, and more complex mechanisms may be involved as well in some (or many) of the recent strong or even unprecedented extremes^16^,^17^,^18^,^19^. Explanations include changes in soil-moisture^17^,^18^, changing tropical Pacific sea surface temperature^20^,^21^, and the potential impact of rapid Arctic warming^19^,^22^,^23^,^24^,^25^.

Coumou *et al*.^19^ showed that the Northern Hemisphere summer jet and associated storm activity have weakened since 1979 and hypothesized that this could lead to more persistent, and therefore more extreme, summer weather. Decreases in summer cyclone activity also lead to a decrease in cloud cover, giving rise to higher maximum temperatures^24^. This weakening in storm activity is seen in future climate model projections as well, linked to rapid warming in the Arctic, but the observed decline is faster than predicted^19^,^26^.

In earlier work, Hoskins and Karoly^27^ and Hoskins and Ambrizzi^28^ demonstrated that Rossby waves within a certain wavenumber range can become effectively trapped in a latitudinal waveguide depending on the structure of the mid-latitude westerlies. Petoukhov *et al*.^29^ showed that if these waves have similar length-scales to those imposed by orographic and thermal forcing, a pronounced amplification of waves can occur due to resonance^1^. The shape of the North-South profile of the zonal-mean westerlies influences the occurrence of this phenomenon of quasi-resonant amplification (QRA)^1^,^2^,^3^,^4^. This profile can change due to changes in the poleward temperature gradient and thus, to the Arctic amplification of greenhouse warming through the thermal wind equation. Tropical expansion can also affect the latitudinal position of the sharpest temperature gradients and latitudinal changes in the land-ocean temperature contrast might play a role as well.

While the underlying mechanisms have been explored in depth elsewhere^1^,^2^,^3^,^4^, the essence of the argument involves the relationship between changing zonal mean temperatures and the strength and position of maxima in the mid-latitude westerly jet. The main condition for resonance is the formation of a zonally-directed waveguide for a particular zonal wavenumber *k*, which depends only on the wavenumber and the shape of the zonal-mean zonal wind (*U*) profile. Such a waveguide is present when a mid-latitude region of positive squared meridional wavenumber *l*^2^ is bounded by latitudes both north and south where *l*^2^ vanishes, inhibiting the dispersion of wave energy and trapping excited planetary waves in the upper troposphere (300–500 mb). This can occur for zonal wavenumbers *k* = 6–8^1^,^2^, with the waveguide found at the equatorward flank of the subtropical jet at latitudes around 30–45°N.

Such conditions are typically associated with a profile for *U* characterized by two maxima in the Northern Hemisphere, i.e. a double jet latitudinal structure. In contrast to a single jet, a double jet regime associated with a profile for *U* is characterized by a confined sub-tropical jet with sharp edges wherein wind speeds change rapidly with latitude^3^. Such sharp sub-tropical jets are highly effective waveguides^30^,^31^, a central requirement for QRA. Especially long-duration resonance events (as e.g. seen during the European and Russian heat waves in 2003 and 2010) have such double jet structures which in turn, through the thermal wind relationships, are characterized by a particular pattern of latitudinal variation in zonal-mean surface and lower tropospheric temperatures^3^. Recent work has reported a clustering of resonance events since 2000 during the satellite reanalysis era (1979-present) that coincides with rapid warming of Arctic surface temperatures, which is suggestive of a possible climate change connection^4^.

Here, we build on recent work by developing an observational temperature-based fingerprint for QRA conditions. We examine, both in long-term historical observations and state-of-the-art (“CMIP5”) climate model simulations, the changes over time in the projection of this fingerprint. Our study focuses on hemispheric-scale trends as it has been shown^1^,^2^ that many recently observed QRA events were hemispheric in nature. A worthwhile extension of the study might focus separately on different (e.g. Atlantic vs. Pacific) sectors of the Northern Hemisphere.

Our approach is conceptually similar^32^,^33^ to statistical downscaling of climate model simulations: We utilize large-scale features of the climate model simulations considered reliable (in this case, meridional temperature gradients) and then exploit a robust empirical relationship that exists between that feature and the response of interest (planetary wave dynamics related to QRA). It is well-documented that climate models reasonably well capture changes in global patterns of surface temperature, which are primarily thermodynamically controlled. In contrast, there is much less confidence in circulation aspects of climate change, which are primarily controlled by dynamics^34^,^35^,^36^. We assume here that even models that don’t correctly simulate certain details of planetary wave dynamics responses^37^— an issue we will examine later– are still likely to get the QRA fingerprint right, allowing us to draw reliable real-world conclusions about how climate change may impact the phenomenon of QRA.

Meridional gradients in lower tropospheric temperatures, as discussed above, imply changes in mid and upper tropospheric zonal wind (*U*) through the thermal wind relationship. Advantages in using the former, rather than latter, as a measure of QRA-favorable conditions are that (a) long-term historical observations of surface temperature are available back through the late 19th century (this is not the case with upper level atmospheric variables) and (b) there is a fairly straightforward and robust impact of anthropogenic climate change on changes in the structure of lower tropospheric temperatures (e.g. via the mechanism of Arctic amplification and/or the enhanced land-ocean temperature contrast).

Using ERA interim reanalysis data and the QRA detection scheme presented in Kornhuber^3^ (see Methods for details) we produced a composite of boreal summer (JJA) zonal mean near-surface (1000 mb) temperature profiles during QRA-favorable time intervals (Fig. 1a). Differencing the QRA-favorable and climatological mean profiles, we define an anomalous zonal mean temperature profile associated with QRA conditions (Fig. 1b). We restrict the profile to the mid-latitude (25–75N) region of interest and center the profile on zero, yielding a “fingerprint” (Fig. 1c) in the zonal mean temperature field associated with QRA-favorable conditions. The fingerprint exhibits negative values in the subtropics, increases to near-neutral values at 40N, a decline toward more negative values through 50N, and pronounced positive values again at higher sub-polar latitudes. While Arctic-amplified warming projects onto this latitudinal anomaly pattern, the fingerprint has considerably more latitudinal structure (including the contribution from an enhanced land/ocean contrast along the Arctic Ocean shore) than is characterized simply by polar amplification alone.

The anomalous meridional temperature gradient associated with the fingerprint (Fig. 1d) is characterized by a large positive peak in the mid-latitude zone of 50–65N, which, via thermal wind, implies a more-pronounced minimum in the zonal mean zonal wind and thus promotes the formation of a double jet. More poleward (i.e. beyond 65N) the reduction in the temperature gradient (and thus a stronger *negative* gradient) implies stronger westerlies in sub-polar regions. These are precisely the conditions that Pethoukov *et al*.^1^ identify as QRA-favorable.

Having defined a fingerprint for QRA-favorable conditions, we next examined mid-latitude (25–75N) zonal mean temperatures from the historical simulations of the Coupled Model Intercomparison Project Phase 5 (CMIP5) experiments^16^ (see Supp Info for details) which comprise *N* = 164 distinct simulations (Table 1) in the anthropogenic+natural “all-forcing” case and *N* = 40 simulations for the “anthropogenic-only” forcing experiments (see Supp. Info.).

The multimodel ensemble zonal mean surface temperatures for the relevant (JJA) season (Fig. 2) show the expected polar amplification of warming, with higher-latitude regions tending to warm more than lower-latitude regions (Fig. 2a). This pattern is expected from the warm-season ice-albedo feedback associated with anthropogenic greenhouse warming, and it is considerably more pronounced in the anthropogenic-only forcing experiments (Fig. 2b) where the complicating effects of volcanic forcing are absent. The full distribution of trends among the multimodel ensemble is shown for the mean extratropical (25–75N average) temperature series (Fig. 2c,d). The series show considerable variability, emphasizing the potentially confounding role of internal variability in identifying clear trends in the zonal mean profiles in individual historical realizations (including the actual observed realization).

The next step in the analysis involves projecting the QRA fingerprint (i.e. Fig. 1c) onto the CMIP5 zonal mean temperature profiles (Fig. 3) using linear regression (see Methods). Examining both the full CMIP5 all-forcing simulations, (Fig. 3a) and anthropogenic-only simulations (Fig. 3b), we observe a large amount of variability among the individual realizations of the multimodel ensemble, again emphasizing the significant role of internal variability. A positive long-term trend is nonetheless evident in most realizations, and is clearly evident in the ensemble means (Fig. 3a,b). This trend is formally independent of global warming, since it reflects a change over time in a relative latitudinal pattern of temperature variation rather than any change in mean hemispheric or global warmth. Comparing the ensemble mean fingerprint series to the ensemble mean extratropical (25–75N) mean temperature for the all-forcing simulations (Fig. 3c) nonetheless reveals a similar long-term increase consistent with polar amplification from anthropogenic warming.

The post-1970 trend of 0.006 units/year in the multimodel mean fingerprint series (Fig. 3c) is highly significant (*p* < 0.0001). Of greater interest, however, is the behavior of individual ensemble members given that the historical changes observed reflect a single realization of internal natural variability. Examining the distribution of trends from the full multimodel ensemble, the median trend lies close to the mean trend, and 68% of the multimodel realizations exhibit a positive trend, which is highly unlikely to have arisen from chance (*p* < 0.0001 based on the null hypothesis of red noise; see Table 1 and Methods). Nonetheless, nearly one third of the realizations thereby do *not* display a positive trend, suggesting that, if the CMIP5 historical simulations are an accurate representation of the distribution of possible historical scenarios, an anthropogenic increase in QRA-favorable conditions is only *likely* (and not *very likely*) in the IPCC lexicon, to be observed in any specific realization of historical temperature variability, including the unique realization that we have actually observed.

For the anthropogenic-only case (Fig. 3d), the post-1970 trend is greater (0.01 units/year), and an increase is found in 88% of the multimodel ensemble members (also significant at the *p* < 0.0001 level; Table 1), nearly breaching the IPCC “very likely” threshold (Table 1). The more dominant trend in this case appears to be a consequence of the absence of volcanic forcing, particularly the absence of the prominent 1982 El Chichon and 1991 Pinatubo eruptions. It is reasonable to conclude that these volcanic events have likely acted to obscure an anthropogenic signal in the QRA fingerprint.

It is noteworthy that the anthropogenic-only ensemble mean QRA fingerprint series exhibits a more monotonic increase through the 1950s and 1960s than the corresponding extratropical mean temperature series (Fig. 3d), the latter of which shows a more pronounced downturn during the 1950–1970 interval of enhanced negative anthropogenic aerosol forcing. This difference in the temporal evolution of the two series ostensibly arises from the fact that anthropogenic aerosol forcing exhibits a regionally heterogeneous spatial pattern of temperature influence, and projects very weakly onto the meridional structure of QRA fingerprint. Thus, it is the more temporally monotonic anthropogenic greenhouse gas forcing that projects primarily onto the QRA fingerprint, and the fingerprint series continues to increase due to greenhouse warming even during a period of pronounced anthropogenic aerosol forcing.

We next project the QRA fingerprint series onto the zonal mean 300 mb zonal velocity (*U*) field for each of the CMIP5 simulations. These projections (Fig. 4) show the expected double jet structure, with a substantial negative zonal wind anomaly in the 50–65N mid-latitude zone and a pronounced positive zonal wind anomaly at higher subpolar latitudes. These features are evident in nearly every simulation for both all-forcing and anthropogenic-only experiments. The precise location and magnitude of the mid-latitude negative peak varies among simulations, and a handful of simulations don’t exhibit the feature at all, consistent with previous work^34^ finding that some models do exhibit biases in upper level winds and especially the bimodality of the jet stream. Such caveats notwithstanding, our results suggest the QRA fingerprint detected in the CMIP5 simulations is indeed associated with a thermal wind-driven anomalous double peak zonal jet structure, consistent with the planetary wave dynamics associated with QRA.

Finally, we apply the QRA fingerprint approach to observational surface temperature data available through 2015 (Fig. 5). As with the CMIP5 historical simulations, we see the expected pattern of polar amplification of warming, regardless of which of three different surface temperature products (GISTEMP, HadCRUT4, and Cowtan & Way) is used (Fig. 5a,b,c). When we project the QRA fingerprint onto the zonal temperature profiles (Fig. 5d,e,f), we observe a significant trend in the QRA fingerprint during the post-1970 interval during which the CMIP5 simulations display a prominent trend. The trend is significant at the *p* = 0.05 level for all three instrumental temperature datasets (Table 1). While the trends in the observational fingerprint series are modestly greater (0.016–0.022 units/year) than those for the CMIP5 multimodel mean series, both are in agreement taking into account respective one sigma ranges (Table 1). The QRA fingerprint series for one of the surface temperature datasets (GISTEMP) exhibits positive values during the early decades of the 20^th^ century that rival recent values. Examining Fig. 5a–c, it is apparent that high-latitude warmth substantially exceeds low-latitude warmth during these decades for GISTEMP, though this is not seen in the other datasets. We suspect that the GISTEMP approach of spatially-interpolating across the Arctic based on what data are available, becomes increasingly susceptible to large sampling uncertainties as data in this region become exceptionally sparse in earlier decades. That could lead to spurious amplification of high-latitude meridional temperature gradients that project substantially onto the QRA fingerprint.

If we restrict the time interval under examination to the period of overlap between the observations and CMIP5 historical simulations that terminates earlier in 2005, we find that the trend in the fingerprint series is statistically significant at the *p* = 0.05 level for only one (GISTEMP) of the three surface temperature datasets (Table 1). Combined with inferences drawn above from the analysis of CMIP5 historical simulations, these observations suggest that the anthropogenic signal in the QRA fingerprint has emerged from the background noise of natural variability only within the past decade or so.

In summary, our analysis of both historical model simulations and observational surface temperature data, strongly suggests that anthropogenic warming is impacting the zonal mean temperature profile in a manner conducive to wave resonance and a consequent increase in persistent weather extremes in the boreal summer. Combined with other additional proposed mechanisms for climate change impacts on extreme weather, this adds to the weight of evidence for a human influence on the occurrence of devastating events such as the 2003 European heat wave, the 2010 Pakistan flood and Russian heat wave, the 2011 Texas heat wave and recent floods in Europe.

## Methods

### QRA Fingerprint

We used the QRA detection scheme of Kornhuber *et al*.^3^ but in this case applied it to near-surface temperature rather than upper level winds, to develop a zonal mean QRA temperature fingerprint. We used the Jun-Aug (JJA) 1979–2015 ERA interim reanalysis (2.5° × 2.5°). Near-surface atmospheric temperature profiles were calculated using z = 1000 mb daily data over the full Northern Hemisphere (0°N–90°N) in 37 steps (2.5°). We focused on long duration (≥10 days) wavenumber 7 events to define QRA-favorable time intervals, and confined zonal-mean profiles to the mid-latitude latitude range (25N–75N) analyzed in previous work^1^,^2^,^3^. Such events have been shown to be closely related to Northern Hemisphere JJA heat extremes^24^. The fingerprint was interpolated to a 5° latitude grid that is consistent with the grid of the model simulations and observational datas (see below) and centered on zero. This yields an 11 element zero-centered row vector that defines the QRA fingerprint.

The projection of the fingerprint was estimated in both CMIP5 simulated (see below) and observational (see below) datasets through linear regression of the 11 element row vectors defined by the individual yearly (JJA) model or observational zonal mean surface temperatures onto the QRA fingerprint defined above.

### CMIP5 Historical Simulations

We used the Coupled Model Intercomparison Project Phase 5 (CMIP5) historical experiment^38^ multimodel ensemble simulations, including both the anthropogenic+natural forced simulations (*N* = 164 realizations; *M* = 48 models) and anthropogenic-only forced simulations (*N* = 40 realizations; *M* = 10 models) spanning 1861–2005 (Table S1). Each physics version of a model was considered a separate model. The analysis was limited to the common time period of overlap for all models and realizations (1861–2005). Those with a start year later than 1861 were not included in the analysis.

We used a simple area-weighted average to create zonal means at a 5° interval.

### Observational Surface Temperatures

We analyzed zonal mean boreal summer (JJA) average temperatures (Surface Air Temperature over land and Sea Surface Temperature over oceans) using each of three alternative datasets: GISTEMP^39^ (1880–2015), HadCRUT4^40^ (1894–2015) and Cowtan & Way^41^ (1894–2015). These three different datasets make alternative assumptions about how to account for historical data gaps, which is particularly important in the Arctic region that is key to this study, due to the large spatial gaps in this region coupled with the amplified high-latitude warming in the Northern Hemisphere in recent decades. The time period of analysis was constrained by continuous data availability for each latitude band. For example, the HadCRUT4 and Cowtan & Way datasets contain at least one grid cell temperature value in each latitude band across the range of interest (25–75N) from 1894 to present. Accordingly, the grid cell coverage is relatively sparse in the early segments of the records and increases toward present day.

As with the model output, we used a simple area-weighted average to create zonal means at a 5° interval.

### Trend Assessment

Linear trends for both simulations and observations were computed based on ordinary least squares (OLS) using the post-1970 interval of dominant anthropogenic forcing.

For the observational series, trends were calculated both for the 1970–2005 (*N* = 36) interval of overlap with the CMIP5 historical simulations, and for the longer 1970–2015 (*N* = 46 years) updated to include the most recent decade of observations. In both cases, *p* values were assessed by taking into account reduced degrees of freedom owing to serial correlation of regression residuals via *N*’ = *N*(1 − *ρ*)/(1 + *ρ*) where *ρ* is the lag-one autocorrelation coefficient of the actual series (Table 1).

For the CMIP5 multimodel mean all-forcing and anthropogenic-only series, trends over 1970–2005 (*N* = 36) and statistical significance were assessed as described above.

We also diagnosed trends for the full CMIP5 multimodel ensemble, assessing the +/−1 sigma range (or more precisely, the 16%ile and 84%ile of the distribution, which is slightly asymmetric with respect to the mean) across the ensemble as reported in Table 1. We also calculated, as a measure of robustness across the multi-model ensemble, the percent of ensemble members (“% > 0” in Table 1) with positive trends and performed Monte Carlo simulations (10,000 realizations) modeling each multi-model ensemble member fingerprint series as AR(1) red noise (preserving the standard deviation and lag-one autocorrelation of the series). Using the distribution among the Monte Carlo surrogates of the 1970–2005 trends across the multimodel ensemble, we then assessed *p* values for the “% > 0” statistic (the minimum possible *p* value calculable using 10,000 surrogates is 10^−4^).

## Additional Information

**How to cite this article:** Mann, M. E. *et al*. Influence of Anthropogenic Climate Change on Planetary Wave Resonance and Extreme Weather Events. *Sci. Rep.* **7**, 45242; doi: 10.1038/srep45242 (2017).

**Publisher's note:** Springer Nature remains neutral with regard to jurisdictional claims in published maps and institutional affiliations.

## Supplementary Material

Supplementary Information

## Figures and Tables

**Figure 1:**
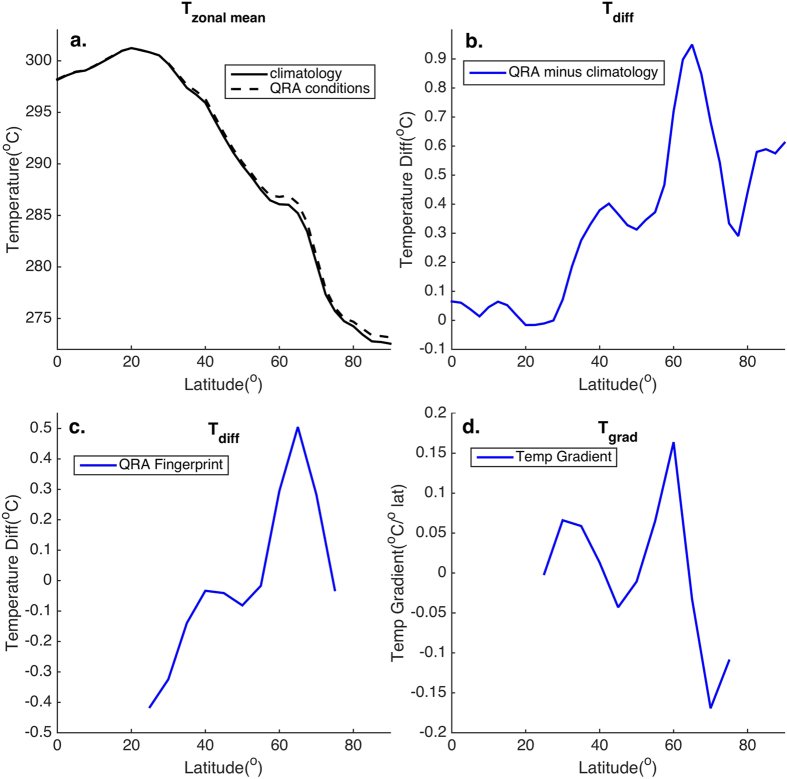
Boreal Summer (JJA) Zonal Mean Temperature Profiles. Shown are (**a**) JJA 1000 mb temperatures from ERA reanalysis data (1979–2015) for both climatological mean and QRA-favorable conditions (at 2.5° latitudinal resolution), (**b**) The difference between the two i.e. the anomalous zonal mean temperature profile associated with QRA-favorable conditions, (**c**) The QRA fingerprint defined as the former quantity, confined to the extatropical region 25–75N, centered on zero, and interpolated onto 5° latitudinal grid commensurate with model simultions, and (**d**) the associated meridional temperature gradient.

**Figure 2:**
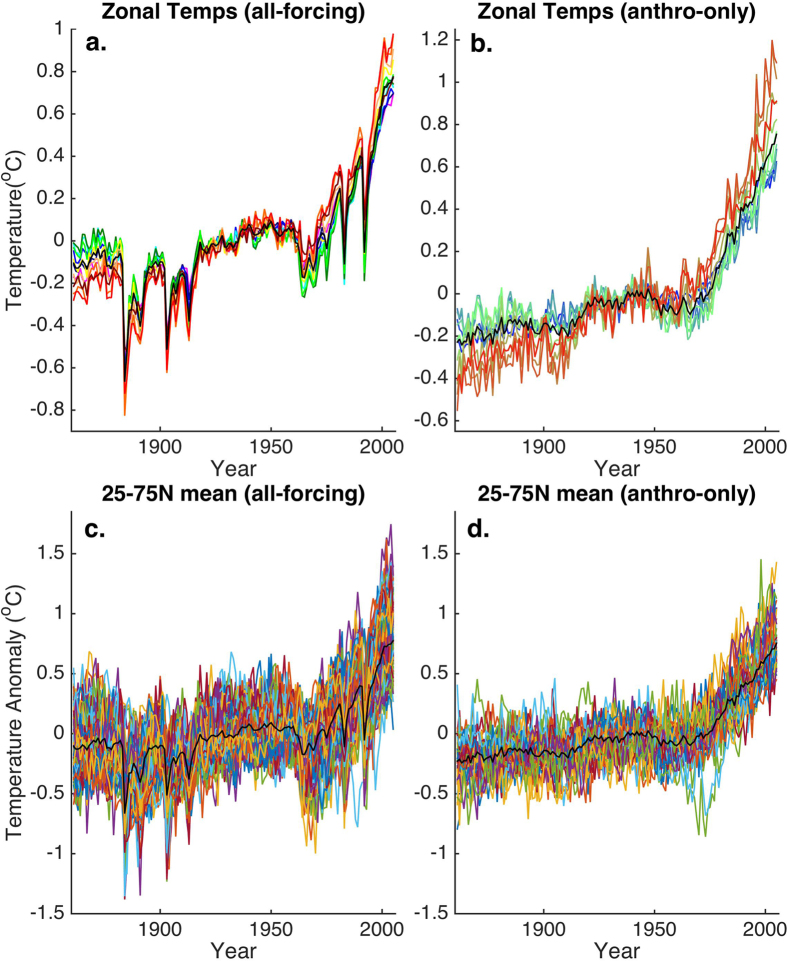
Zonal mean temperatures for 5 degree bands from 25–75N in the CMIP5 historical simulations. Shown are the multimodel means for the (**a**) all-forcing and (**b**) anthropogenic-only simulations. Rainbow scale is used to denote increasing latitude from 25N (violet) to 75N (red). The extratropical 25–75N mean series is shown for comparison (black). Shown also for both the (**c**) all-forcing and (**d**) anthropogenic-only simulations is the extratropical mean series for each member of the multimodel ensemble (colored curves) along with the multimodel mean (black) series.

**Figure 3:**
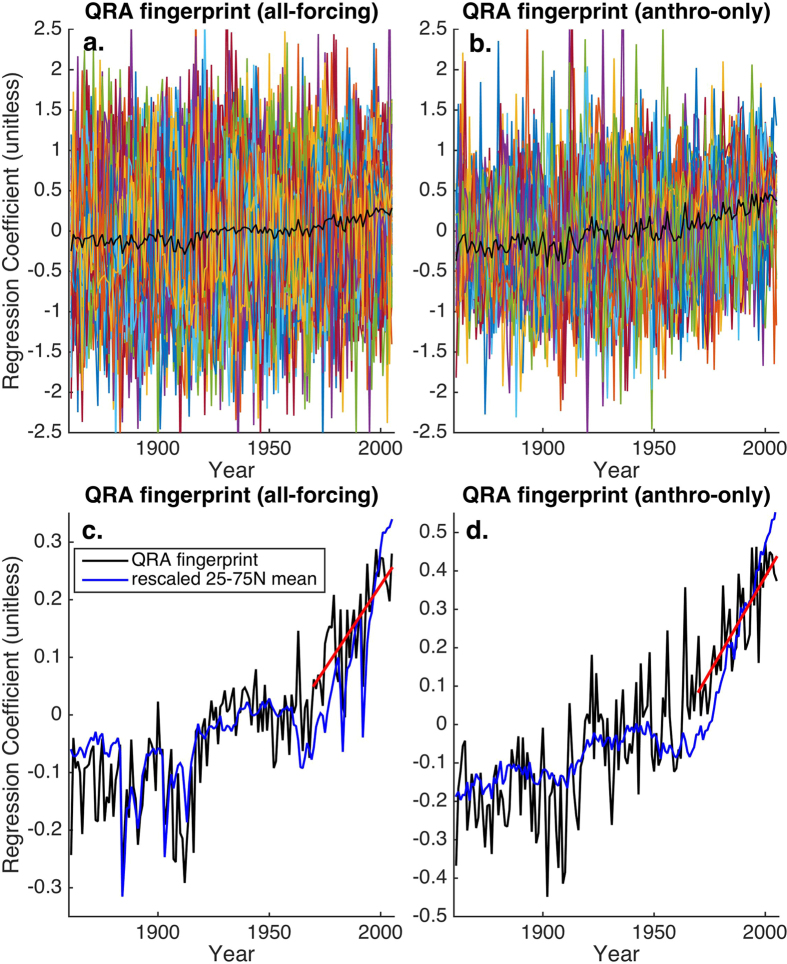
QRA Fingerprint Series. Shown for both the (**a**) all-forcing and (**b**) anthropogenic-only simulations is the QRA series for each member of the multimodel ensemble (colored curves) along with the multimodel mean (black) series. The corresponding multimodel mean QRA series are shown along with the exatropical mean temperatures series for the (**c**) all forcing and (**d**) anthropogenic-only simulations, along with the linear trend over 1970–2005 (red line).

**Figure 4:**
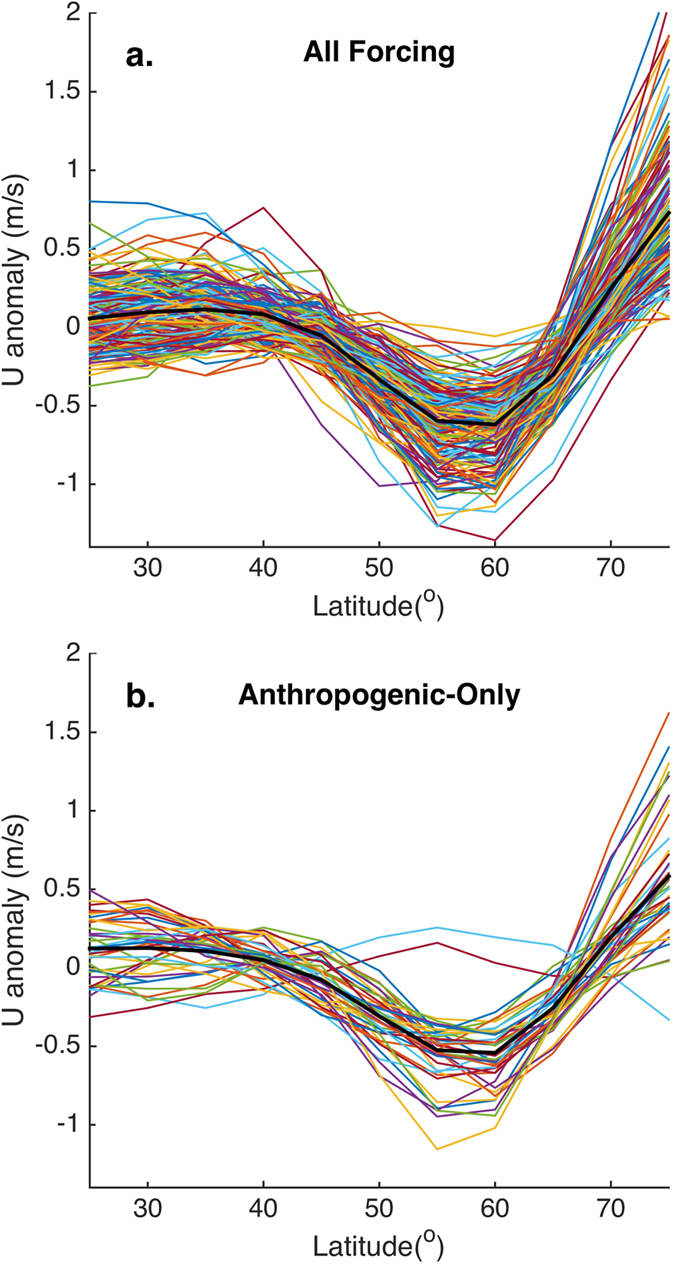
Projection of QRA Fingerprint Series onto 300 mb Zonal Wind (U) in individual CMIP5 historical Simulations (colored curves) and averaged over ensemble (black). Shown are results for (**a**) all-forcing and (**b**) anthropogenic-only forcing experiments.

**Figure 5:**
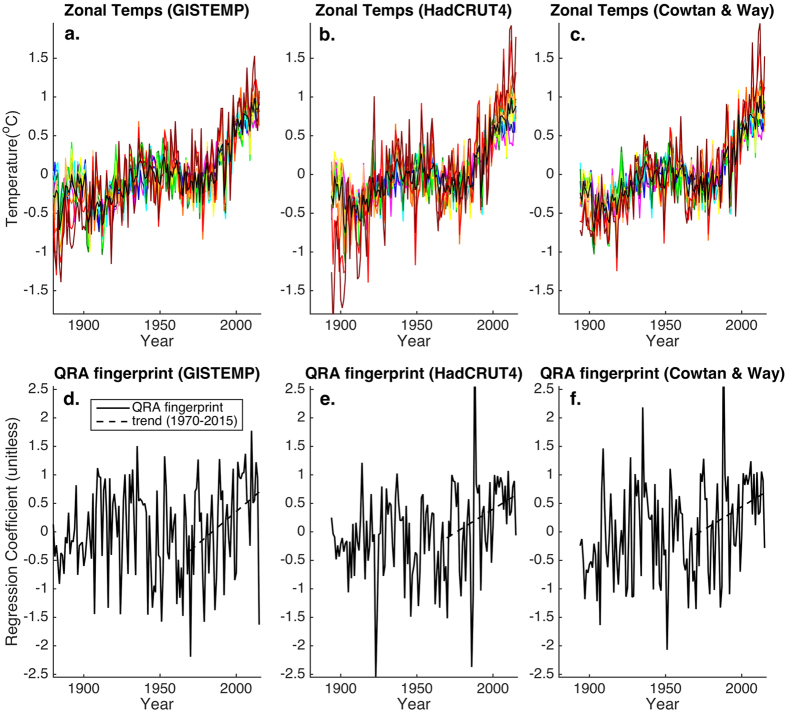
Observational Series. Shown are the zonal mean temperatures for 5 degree bands from 25–75N (colors—same conventions as in Fig. 2a) and the extratropical 2–75N mean series (black) for (**a**) GISTEMP, (**b**) HadCRUT4, and (**c**) Cowtan & Way instrumental temperature series. Shown are the corresponding QRA fingerprint series (**d**–**f**) along with linear trend from 1970–2015 (black dashed).

**Table 1:** Post-1970 Trend in QRA fingerprint series.

CMIP5 All-Forcing (1970–2005)	Slope (units/yr)	% > 0	*p*	
Multimodel Mean	0.006		<*0.0001*	
Multimodel Ensemble (−1σ, +1σ)	(−0.005, 0.017)	68	<*0.0001*	
CMIP5 Anthropogenic-Only (1970–2005)				
Multimodel Mean	0.010			
Multimodel Ensemble (−1σ, +1σ)	(0.0009, 0.020)	88	<*0.0001*	
**Observations (1970–2015)**	**Slope (units/yr)**	**t**	***dof***	***p***
GISTEMP	0.022 ± 0.01	2.35	35	0.01
HadCRUT4	0.017 ± 0.01	1.68	34	0.05
Cowtan & Way	0.016 ± 0.01	1.68	37	0.05
**Observations (1970–2005)**	**Slope (units/yr)**	**t**	***dof***	***p***
GISTEMP	0.028 ± 0.014	2.03	22	0.03
HadCRUT4	0.019 ± 0.016	1.16	25	0.13
Cowtan & Way	0.018 ± 0.015	1.17	27	0.13
