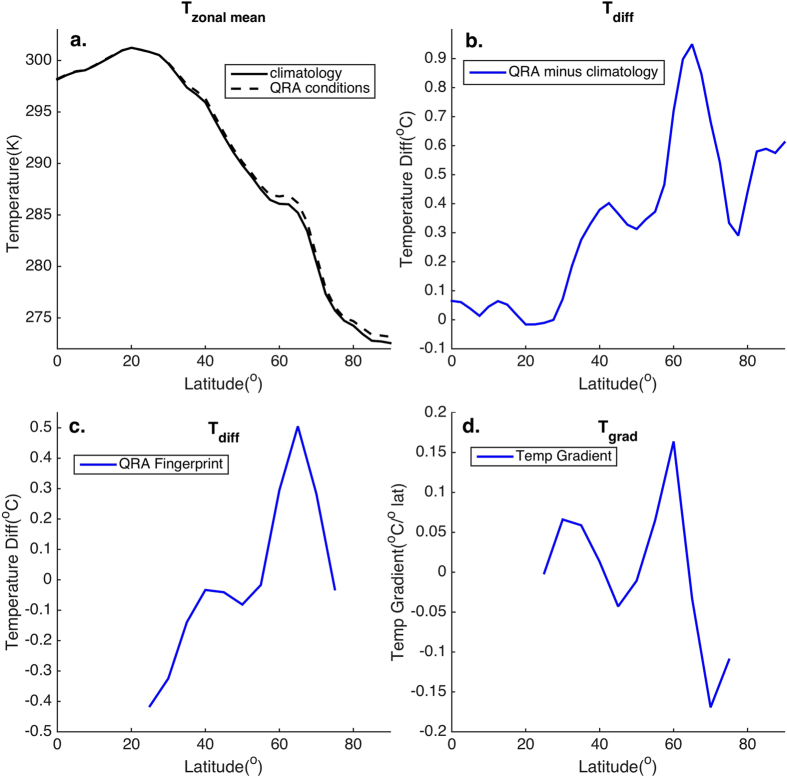# Corrigendum: Influence of Anthropogenic Climate Change on Planetary Wave Resonance and Extreme Weather Events

**DOI:** 10.1038/srep46822

**Published:** 2017-05-26

**Authors:** Michael E. Mann, Stefan Rahmstorf, Kai Kornhuber, Byron A. Steinman, Sonya K. Miller, Dim Coumou

Scientific Reports 7: Article number: 45242; 10.1038/srep45242 published online: 03 27 2017; updated: 05 26 2017.

This Article contains an error in Figure 1a, where the y-axis ‘Temperature (K)’ is incorrectly labelled as ‘Temperature (°C)’. The correct Figure 1 appears below.

## Figures and Tables

**Figure 1:**