# Supplementary material for: Influence of Anthropogenic Climate Change on Planetary Wave Resonance and Extreme Weather Events
Source: Sci Rep. 2017 Mar 27;7:45242. doi: 10.1038/srep45242 (PMC5366916; doi:10.1038/srep45242)
Supplement: Supplementary Information [file srep45242-s1.docx]

**Supplementary Information**

**Table S1**. CMIP5 Climate Model Simulations

| Model | Number of Realizations | 1^st^ *and* 2^nd^ aerosol indirect effects |
| --- | --- | --- |
| ACCESS1-0 | 1 | 1 |
| ACCESS1-3 | 3 | 1 |
| bcc-csm1-1 | 3 | 0 |
| bcc-csm1-1-m | 3 | 0 |
| BNU-ESM | 1 | 0 |
| CanESM2 | 5 | 0 |
| CCSM4 | 6 | 0 |
| CESM1-BGC | 1 | 0 |
| CESM1-CAM5 | 3 | 1 |
| CESM1-FASTCHEM | 3 | 0 |
| CESM1-WACCM | 1 | 0 |
| CMCC-CESM | 1 | 0 |
| CMCC-CM | 1 | 0 |
| CMCC-CMS | 1 | 0 |
| CNRM-CM5 | 10 | 0 |
| CNRM-CM5-2 | 1 | 0 |
| CSIRO-Mk3-6-0 | 10 | 1 |
| FGOALS-g2 | 4 | 1 |
| FIO-ESM | 3 | 0 |
| GFDL-CM2.1 | 10 | 0 |
| GFDL-CM3 | 5 | 1 |
| GFDL-ESM2G | 1 | 0 |
| GFDL-ESM2M | 1 | 0 |
| GISS-E2-H p1 | 6 | 0 |
| GISS-E2-H p2 | 5 | 0 |
| GISS-E2-H p3 | 6 | 0 |
| GISS-E2-H-CC | 1 | 0 |
| GISS-E2-R p1 | 6 | 0 |
| GISS-E2-R p2 | 6 | 0 |
| GISS-E2-R p3 | 6 | 0 |
| GISS-E2-R p100 | 6 | 0 |
| GISS-E2-R-CC | 1 | 0 |
| HadCM3 | 10 | 0 |
| HadGEM2-AO | 1 | 1 |
| HadGEM2-CC | 1 | 1 |
| HadGEM2-ES | 5 | 1 |
| INMCM4 | 1 | 0 |
| IPSL-CM5A-LR | 6 | 0 |
| IPSL-CM5A-MR | 3 | 0 |
| IPSL-CM5B-LR | 1 | 0 |
| MIROC-ESM | 3 | 1 |
| MIROC-ESM-CHEM | 1 | 1 |
| MPI-ESM-LR | 2 | 0 |
| MRI-CGCM3 p1 | 3 | 1 |
| MRI-CGCM3 p2 | 1 | 1 |
| MRI-ESM1 | 1 | 1 |
| NorESM1-M | 3 | 1 |
| NorESM1-ME | 1 | 1 |
| Anthropogenic Only Simulation | | |
| CCSM4 p11 | 4 | – |
| CESM1-CAM5 p11 | 3 | – |
| CNRM-CM5 | 10 | – |
| GFDL-CM3 p2 | 3 | – |
| GFDL-ESM2M p2 | 1 | – |
| GISS-E2-H p109 | 3 | – |
| GISS-E2-H p309 | 3 | – |
| GISS-E2-R p109 | 5 | – |
| GISS-E2-R p309 | 5 | – |
| IPSL-CM5A-LR p2 | 3 | – |
